# Complete genome sequence of *Ilyobacter polytropus* type strain (CuHbu1^T^)

**DOI:** 10.4056/sigs.1273360

**Published:** 2010-12-15

**Authors:** Johannes Sikorski, Olga Chertkov, Alla Lapidus, Matt Nolan, Susan Lucas, Tijana Glavina Del Rio, Hope Tice, Jan-Fang Cheng, Roxane Tapia, Cliff Han, Lynne Goodwin, Sam Pitluck, Konstantinos Liolios, Natalia Ivanova, Konstantinos Mavromatis, Natalia Mikhailova, Amrita Pati, Amy Chen, Krishna Palaniappan, Miriam Land, Loren Hauser, Yun-Juan Chang, Cynthia D. Jeffries, Evelyne Brambilla, Montri Yasawong, Manfred Rohde, Rüdiger Pukall, Stefan Spring, Markus Göker, Tanja Woyke, James Bristow, Jonathan A. Eisen, Victor Markowitz, Philip Hugenholtz, Nikos C. Kyrpides, Hans-Peter Klenk

**Affiliations:** 1 DSMZ - German Collection of Microorganisms and Cell Cultures GmbH, Braunschweig, Germany; 2 DOE Joint Genome Institute, Walnut Creek, California, USA; 3 Los Alamos National Laboratory, Bioscience Division, Los Alamos, New Mexico, USA; 4 Biological Data Management and Technology Center, Lawrence Berkeley National Laboratory, Berkeley, California, USA; 5 Oak Ridge National Laboratory, Oak Ridge, Tennessee, USA; 6 HZI – Helmholtz Centre for Infection Research, Braunschweig, Germany; 7 University of California Davis Genome Center, Davis, California, USA

**Keywords:** strictly anaerobic, none-motile, Gram-negative, 3-hydroxybutyrate, mesophilic, chemoorganotrophic, *Fusobacteriaceae*, GEBA

## Abstract

*Ilyobacter polytropus* Stieb and Schink 1984 is the type species of the genus *Ilyobacter*, which belongs to the fusobacterial family *Fusobacteriaceae*. The species is of interest because its members are able to ferment quite a number of sugars and organic acids. *I. polytropus* has a broad versatility in using various fermentation pathways. Also, its members do not degrade poly-*β*-hydroxybutyrate but only the monomeric 3-hydroxybutyrate. This is the first completed genome sequence of a member of the genus *Ilyobacter* and the second sequence from the family *Fusobacteriaceae*. The 3,132,314 bp long genome with its 2,934 protein-coding and 108 RNA genes consists of two chromosomes (2 and 1 Mbp long) and one plasmid, and is a part of the *** G****enomic* *** E****ncyclopedia of* *** B****acteria and* *** A****rchaea * project.

## Introduction

Strain CuHbu1^T^ (= DSM 2926 = ATCC 51220 = LMG 16218) is the type strain of *I. polytropus*, which is the type species of the genus *Ilyobacter* [1,2]. Currently, there are four species placed in the genus *Ilyobacter* [1]. The generic name derives from the Greek word ‘*ilus*’ meaning ‘mud’ and the Neo-Latin word ‘*bacter*’ meaning ‘a rod’, referring to a mud-inhabiting rod [2]. The species epithet is derived from the Neo-Latin word ‘*polytropus*’ meaning ‘versatile’, referring to metabolic versatility of the species [2]. *I. polytropus* strain CuHbu1^T^ was isolated from marine anoxic mud in Cuxhaven, Germany, and described by Stieb and Schink in 1984 [2]. No further isolates have been obtained for *I. polytropus*. Members of the genus *Ilyobacter* were isolated from anoxic marine sediments in Germany [2], Italy [3,4] and of estuarine origin [5]. Here we present a summary classification and a set of features for *I. polytropus* CuHbu1^T^, together with the description of the complete genomic sequencing and annotation.

## Classification and features

**Figure 1:**
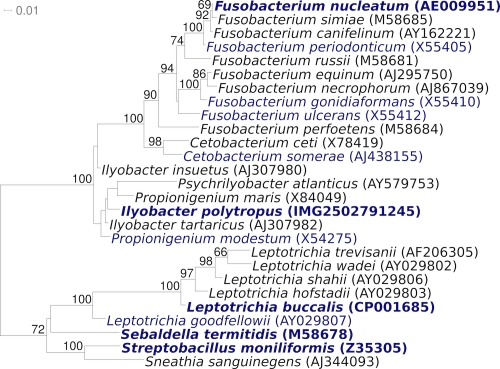
Phylogenetic tree highlighting the position of *I. polytropus* CuHbu1^T^ relative to the other type strains within the family *Fusobacteriaceae*. The tree was inferred from 1,399 aligned characters [7,8] of the 16S rRNA gene sequence under the maximum likelihood criterion [9] and rooted in accordance with the current taxonomy. The branches are scaled in terms of the expected number of substitutions per site. Numbers above branches are support values from 900 bootstrap replicates [10] if larger than 60%. Lineages with type strain genome sequencing projects registered in GOLD [11] are shown in blue, published genomes in bold [12-15]. Note that *Ilyobacter* appears as polyphyletic in the tree [16], but none of the relevant branches obtains any bootstrap support. Thus, the current classification is not in significant conflict with our phylogenetic analysis.

The 16S rRNA gene sequence of *I. polytropus* shares the highest degree of sequence similarity with the type strains of the other two members of the genus, *I. insuentus* (97.3%) and *I. tartaricus* (98.3%), the latter was isolated from anoxic marine sediment of Canal Grande and Rio Martin in Venice, Italy. The degree of sequence identity with the type strains of the other members of the family *Fusobacteriaceae* varies between 89.5% and 97.8%, with *Propionigenium modestum* as most similar species [6] (Figure 1). The genome survey sequence database (gss) contains the 16S rRNA gene sequence of human gut metagenome clone 5192b-5192b-A-con-04 (FI579563) as the best hit, which is 91% identical to the 16S rRNA gene sequence of strain CuHbu1^T^. No phylotypes from environmental samples database (env_nt) could be linked to the species *I. polytropus* or even the genus *Ilyobacter*, indicating a rather rare occurrence of these in the habitats screened so far (as of October 2010). A representative genomic 16S rRNA sequence of *I. polytropus* was compared using NCBI BLAST under default values (e.g., considering only the best 250 hits) with the most recent release of the Greengenes database [17] and the relative frequencies of taxa and keywords, weighted by BLAST scores, of taxa and keywords were determined. The four most frequent genera were *Fusobacterium* (70.2%), *Ilyobacter* (13.8%), *Propionigenium* (12.4%) and *Clostridium* (3.6%). Regarding hits to sequences from other members of the genus, the average identity within HSPs (high-scoring segment pairs) was 96.4%, whereas the average coverage by HSPs was 98.4%. The species yielding the highest score was *I. tartaricus*. The five most frequent keywords within the labels of environmental samples which yielded hits were 'microbiome' (6.5%), 'fecal' (6.1%), 'feces' (5.7%), 'calves/microorganisms/neonatal/shedding' (5.5%) and 'evolution/gut/mammals/microbes' (2.5%). These keywords suggest further, animal associated habitats for *I. polytropus*, beyond the anaerobic muds of marine origin as stated in the original description [2]. Environmental samples which yielded hits of a higher score than the highest scoring species were not found.

Figure 1 shows the phylogenetic neighborhood of *I. polytropus* CuHbu1^T^ in a 16S rRNA based tree. The sequences of the eight 16S rRNA gene copies in the genome of *I. polytropus* differ from each other by up to three nucleotides, and differ by up to three nucleotides from the previously published 16S rRNA sequence (AJ307981), which contains two ambiguous base calls.

**Figure 2:**
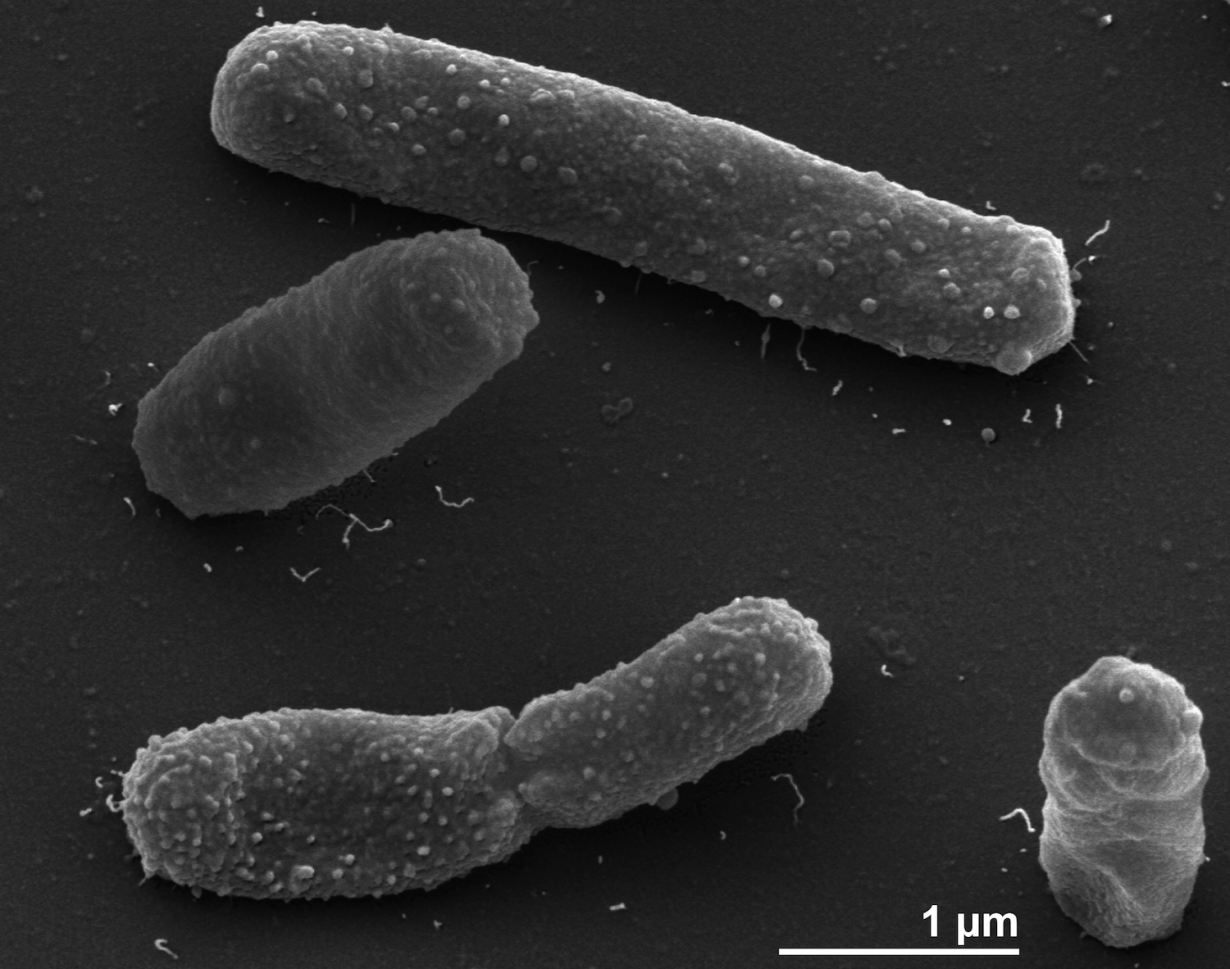
Scanning electron micrograph of *I. polytropus* CuHbu1^T^

The cells of *I. polytropus* are generally rod-shaped (0.7×1.5-3.0 µm) with rounded ends (Figure 2). Cells of *I. polytropus* show irregularly elongated rods, when grown on glucose and fructosecontaining media [2]. The cells are usually arranged in pairs or chains [2]. *I. polytropus* is a Gram-negative and non spore-forming bacterium (Table 1). The organism is nonmotile and no flagellar genes have been found in the genome If at all, active movement by twitching motility could be possible, as some genes related to this phenotype were identified (this paper, see below). Interestingly, the original description states that “the originally motile rods lost motility after several transfers” [2]. The organism is a strictly anaerobic chemoorganotroph [2]. *I. polytropus* requires 1% NaCl in media for good growth [25]. The selective medium for *I. polytropus* is a NaCl-containing mineral media, which contains 3-hydroxybutyrate as a sole carbon and energy source [2]. The organism also grows in salt water medium or brackish water medium containing 1% NaCl and 0.15% MgCl_2_^.^6H_2_O [2]. Vitamins are not required in the enrichment media for at least five subsequent transfers [2]. Phosphate (up to 50 mM) does not inhibit growth of *I. polytropus*, when grown on 3-hydroxybutyrate [2]. The temperature range for growth is between 10°C and 35°C, with an optimum at 30°C [2]. The organism does not grow at 4°C or at 40°C [2]. The pH range for growth is 6.5-8.5, with an optimum at pH 7.0-7.5 [2]. No cytochromes are detected from *I. polytropus* [2]. *I. polytropus* is able to utilize 3-hydroxybutyrate, crotonate, glycerol, pyruvate, citrate, oxaloacetate, glucose, fructose, malate and fumarate and to ferment a variety of sugars and organic acids [2]. The organism does not utilize lactose, sucrose, mannitol, sorbitol, xylitol, 1,2-butanediol, 1,3-butanediol, 2,3-butanediol, maltose, arabinose, cellobiose, mannose, melezitose, raffinose, sorbose, rhamnose, trehalose, xylose, acetone, diacetyl acetoin, acetoacetyl ethylester, acetoacetyl amide, peptone, casamino acids, yeast extract, glyoxylate, glycolate, lactate, succinate, L-tartrate, poly-*β*-hydroxybutyrate, starch, methanol plus acetate and formate plus acetate [2]. *I. polytropus* is able to ferment 3-hydroxybutyrate and crotonate to acetate and butyrate [2]. Glycerol is fermented to 1,3-propanediol and 3-hydroxypropionate [2]. Malate and fumarate are fermented to acetate, formate and propionate [2]. *I. polytropus* is able to ferment glucose and fructose to acetate, formate and ethanol [2]. The organism does not reduce sulfate, sulfur, thiosulfate and nitrate [2]. *I. polytropus* grows in mineral media with a reductant [2]. It does not hydrolyze gelatin or urea and does not produce indole [2]. *I. polytropus* shows acetate kinase, phosphate acetyl transferase and hydrogenase activities, which are sufficient for involvement in dissimilatory metabolism [2]. Also, pyruvate formate lyase activity was shown in crude cell extracts, however, activity was extremely low and ascribed to a potential instability of this enzyme if traces of oxygen are present during the enzyme activity measurement [2]. *I. polytropus* maintains its energy metabolism exclusively by substrate-linked phosphorylation reactions [2]. *I. polytropus* differs from other anaerobes because the organism exhibits broad versatility in its use of various fermentation pathways. However, pathway regulation was reported as enigmatic because neither propionate nor butyrate were formed during glucose or fructose fermentation, although the necessary enzymes are present [2]. *I. polytropus* is of ecological interest because the organism does not degrade poly-*β*-hydroxybutyrate but only the monomeric of 3- hydroxybutyrate [2]. Metabolism of the polymer appears to be confined to aerobic microbial communities [26].

**Table 1:** Classification and general features of *I. polytropus* CuHbu1^T^ according to the MIGS recommendations [18].

**MIGS ID**	**Property**	**Term**	**Evidence code**
	Current classification	Domain *Bacteria*	TAS [19]
		Phylum “*Fusobacteria*”	TAS [20,21]
		Class “*Fusobacteria*”	TAS [20]
		Order “*Fusobacteriales*”	TAS [20]
		Family “*Fusobacteriaceae*”	TAS [20]
		Genus *Ilyobacter*	TAS [2,22]
		Species *Ilyobacter polytropus*	TAS [2,22]
		Type strain CuHbu1	TAS [2]
	Gram stain	negative	TAS [2]
	Cell shape	rod-shaped with rounded ends, single or in pairs	TAS [2]
	Motility	non-motile	TAS [2]
	Sporulation	none	TAS [2]
	Temperature range	10°C–35°C	TAS [2]
	Optimum temperature	30°C	TAS [2]
	Salinity	1% NaCl	TAS [2]
MIGS-22	Oxygen requirement	strictly anaerobic	TAS [2]
	Carbon source	carbohydrates	TAS [2]
	Energy source	chemoorganotroph	TAS [2]
MIGS-6	Habitat	marine anoxic mud	TAS [2]
MIGS-15	Biotic relationship	free-living	NAS
MIGS-14	Pathogenicity	none	NAS
	Biosafety level	1	TAS [23]
	Isolation	marine anoxic mud	TAS [2]
MIGS-4	Geographic location	Cuxhaven, Germany	TAS [2]
MIGS-5	Sample collection time	1983 or before	TAS [2]
MIGS-4.1	Latitude	53.87	NAS
MIGS-4.2	Longitude	8.69	NAS
MIGS-4.3	Depth	not reported	
MIGS-4.4	Altitude	sea level	NAS

Evidence codes - IDA: Inferred from Direct Assay (first time in publication); TAS: Traceable Author Statement (i.e., a direct report exists in the literature); NAS: Non-traceable Author Statement (i.e., not directly observed for the living, isolated sample, but based on a generally accepted property for the species, or anecdotal evidence). These evidence codes are from of the Gene Ontology project [24]. If the evidence code is IDA, then the property was directly observed by one of the authors or an expert mentioned in the acknowledgements.

### Chemotaxonomy

No chemotaxonomic data are currently available for *I. polytropus* or for the genus *Ilyobacter*.

## Genome sequencing and annotation

### Genome project history

This organism was selected for sequencing on the basis of its phylogenetic position [27], and is part of the *** G****enomic* *** E****ncyclopedia of* *** B****acteria and* *** A****rchaea * project [28]. The genome project is deposited in the Genome OnLine Database [11,29] and the complete genome sequence is deposited in GenBank. Sequencing, finishing and annotation were performed by the DOE Joint Genome Institute (JGI). A summary of the project information is shown in Table 2.

**Table 2:** Genome sequencing project information

**MIGS ID**	**Property**	**Term**
MIGS-31	Finishing quality	Finished
MIGS-28	Libraries used	Tree genomic libraries: one 454 pyrosequence standard library, one 454 PE library (14 kb insert size), one Illumina library
MIGS-29	Sequencing platforms	Illumina GAii, 454 GS FLX Titanium
MIGS-31.2	Sequencing coverage	124.8 × Illumina; 91.0 × pyrosequence
MIGS-30	Assemblers	Newbler version 2.0.00.20- PostRelease-10-28-2008-g-3.4.6, phrap
MIGS-32	Gene calling method	Prodigal 1.4, GenePRIMP
	INSDC ID	CP002281 chromosome I CP002282 chromosome II CP002283 plasmid
	Genbank Date of Release	November 1, 2010
	GOLD ID	Gc01413
	NCBI project ID	32577
	Database: IMG-GEBA	2503538000
MIGS-13	Source material identifier	DSM 2926
	Project relevance	Tree of Life, GEBA

### Growth conditions and DNA isolation

*I. polytropus* CuHbu1^T^, DSM 2926, was grown anaerobically in medium 314 (*Ilyobacter polytropus* medium) [30] at 30°C. DNA was isolated from 0.5-1 g of cell paste using MasterPure Gram-positive DNA purification kit (Epicentre MGP04100) following the standard protocol as recommended by the manufacturer, with modification st/LALM for cell lysis as described in Wu *et al*. [28].

### Genome sequencing and assembly

The genome was sequenced using a combination of Illumina and 454 sequencing platforms. All general aspects of library construction and sequencing can be found at the JGI website [31]. Pyrosequencing reads were assembled using the Newbler assembler version 2.0.00.20-PostRelease-10-28-2008-g++-3.4.6 (Roche). The initial Newbler assembly consisting of 85 contigs in 1 scaffold was converted into a phrap assembly by [32] making fake reads from the consensus, to collect the read pairs in the 454 paired end library. Illumina GAii sequencing data (387 Mb) was assembled with Velvet [33] and the consensus sequences were shredded into 1.5 kb overlapped fake reads and assembled together with the 454 data. The 454 draft assembly was based on 284.7 Mb 454 draft data and all of the 454 paired end data. Newbler parameters are -consed -a 50 -l 350 -g -m -ml 20.

The Phred/Phrap/Consed software package [32] was used for sequence assembly and quality assessment in the subsequent finishing process. After the shotgun stage, reads were assembled with parallel phrap (High Performance Software, LLC). Possible mis-assemblies were corrected with gapResolution [31], Dupfinisher, or sequencing cloned bridging PCR fragments with subcloning or transposon bombing (Epicentre Biotechnologies, Madison, WI) [34]. Gaps between contigs were closed by editing in Consed, by PCR and by Bubble PCR primer walks (J.-F.Chang, unpublished). A total of 719 additional reactions were necessary to close gaps and to raise the quality of the finished sequence. Illumina reads were also used to correct potential base errors and increase consensus quality using a software Polisher developed at JGI [35]. The error rate of the completed genome sequence is less than 1 in 100,000. Together, the combination of the Illumina and 454 sequencing platforms provided 215.8 × coverage of the genome. The final assembly contained 656,481 pyrosequence and 10,750,000 Illumina reads

### Genome annotation

Genes were identified using Prodigal [36] as part of the Oak Ridge National Laboratory genome annotation pipeline, followed by a round of manual curation using the JGI GenePRIMP pipeline [37]. The predicted CDSs were translated and used to search the National Center for Biotechnology Information (NCBI) nonredundant database, UniProt, TIGR-Fam, Pfam, PRIAM, KEGG, COG, and InterPro databases. Additional gene prediction analysis and functional annotation was performed within the Integrated Microbial Genomes - Expert Review (IMG-ER) platform [38].

## Genome properties

The genome consists of a 2,046,464 bp long chromosome I with a GC content of 35%, a 961,624 bp long chromosome II with 34% GC content, and a 124,226 bp long plasmid with 32% GC content (Table 3 and Figure 3a, Figure 3b, and Figure 3c). Of the 3,042 genes predicted, 2,934 were protein-coding genes, and 108 RNAs; 108 pseudogenes were also identified. The majority of the protein-coding genes (73.3%) were assigned with a putative function while the remaining ones were annotated as hypothetical proteins. The distribution of genes into COGs functional categories is presented in Table 4.

**Table 3:** Genome Statistics

**Attribute**	**Value**	**% of Total**
Genome size (bp)	3,132,314	100.00%
DNA Coding region (bp)	2,690,412	85.89%
DNA G+C content (bp)	1,076,435	34.37%
Number of replicons	3	
Extrachromosomal elements	1	
Total genes	3,042	100.00%
RNA genes	108	3.55%
rRNA operons	8	
Protein-coding genes	2,934	96.45%
Pseudo genes	54	1.78%
Genes with function prediction	2,230	73.31%
Genes in paralog clusters	676	22.22%
Genes assigned to COGs	2,283	75.05%
Genes assigned Pfam domains	2,359	77.55%
Genes with signal peptides	719	23.64%
Genes with transmembrane helices	638	20.97%
CRISPR repeats	1	

**Figure 3a:**
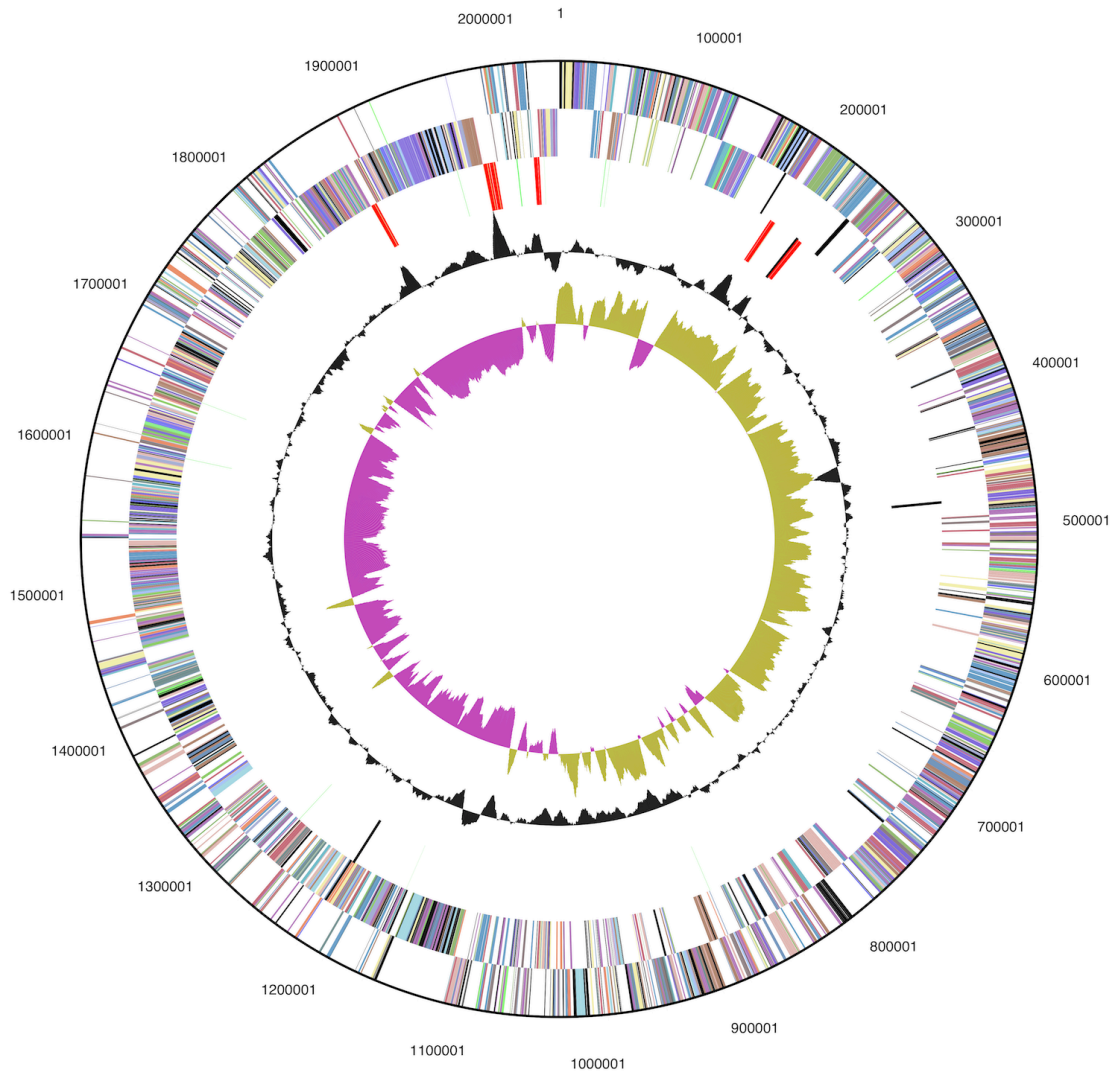
Graphical circular map of chromosome I. From outside to the center: Genes on forward strand (color by COG categories), Genes on reverse strand (color by COG categories), RNA genes (tRNAs green, rRNAs red, other RNAs black), GC content, GC skew.

**Figure 3b:**
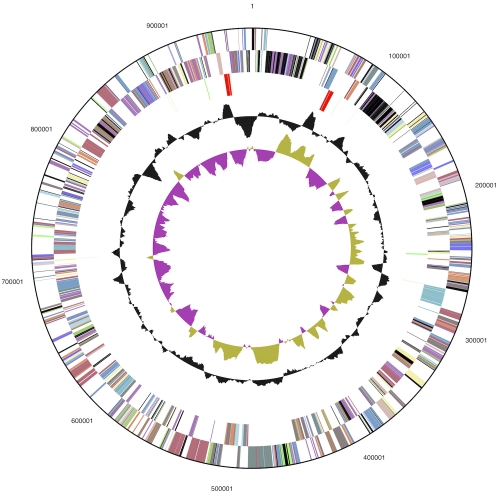
Graphical circular map of chromosome II. From outside to the center: Genes on forward strand (color by COG categories), Genes on reverse strand (color by COG categories), RNA genes (tRNAs green, rRNAs red, other RNAs black), GC content, GC skew. Chromosome II was identified as a chromosome due to its two 16S rRNA gene copies.

**Figure 3c:**
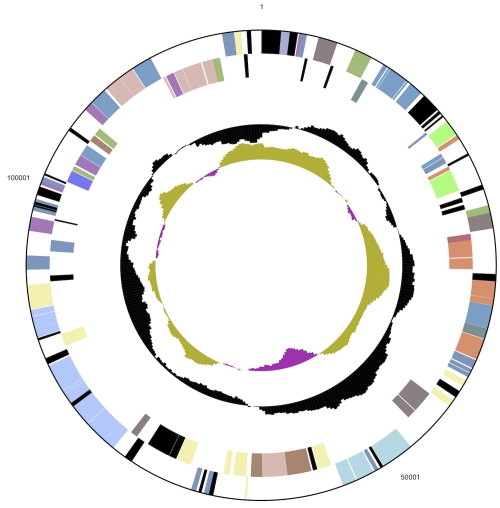
Graphical circular map of the plasmid. From outside to the center: Genes on forward strand (color by COG categories), Genes on reverse strand (color by COG categories), RNA genes (tRNAs green, rRNAs red, other RNAs black), GC content, GC skew. Chromosome II was identified as a chromosome due to its two 16S rRNA gene copies.

**Table 4:** Number of genes associated with the general COG functional categories

**Code**	**value**	**% age**	**Description**
J	146	5.8	Translation, ribosomal structure and biogenesis
A	0	0.0	RNA processing and modification
K	152	6.1	Transcription
L	124	5.0	Replication, recombination and repair
B	1	0.0	Chromatin structure and dynamics
D	27	1.1	Cell cycle control, cell division, chromosome partitioning
Y	0	0.0	Nuclear structure
V	43	1.7	Defense mechanisms
T	140	5.6	Signal transduction mechanisms
M	161	6.4	Cell wall/membrane/envelope biogenesis
N	21	0.8	Cell motility
Z	0	0.0	Cytoskeleton
W	0	0.0	Extracellular structures
U	61	2.4	Intracellular trafficking, secretion, and vesicular transport
O	69	2.8	Posttranslational modification, protein turnover, chaperones
C	244	9.7	Energy production and conversion
G	151	6.0	Carbohydrate transport and metabolism
E	245	9.8	Amino acid transport and metabolism
F	73	2.9	Nucleotide transport and metabolism
H	134	5.4	Coenzyme transport and metabolism
I	72	2.9	Lipid transport and metabolism
P	104	4.2	Inorganic ion transport and metabolism
Q	38	1.5	Secondary metabolites biosynthesis, transport and catabolism
R	311	12.4	General function prediction only
S	190	7.6	Function unknown
-	759	25.0	Not in COGs
